# Supplementary material for: Robustness of genetic diversity measures under spatial sampling and a new frequency-independent measure
Source: PeerJ. 2023 Sep 18;11:e16027. doi: 10.7717/peerj.16027 (PMC10512937; doi:10.7717/peerj.16027)
Supplement: Supplemental Information 1 [file peerj-11-16027-s001.docx]

**Supplemental information**

**Appendix S1. Proof.**

Assumption 1: Simple IBD assumption

Assume *L* location points in which individuals of a taxon live (*L* > 1). The space on which the locations exist can be arbitrary as long as a distance between two locations can be defined in real numbers. The distance between location *i* and *j* is denoted as *d_ij_*, and *D* is the set of all the distances between two locations. Obviously, *D* is a totally ordered set. The substitution rate between two sequences sampled from location *i* and *j* is denoted as *f*(*d_ij_*) + *μ_i_* + *μ_j_*. Here, *μ_i_* and *μ_j_* are independent random variables with the expected value 0 that stand for the effect of mutation within a location on the substitution rate, and *f*(*x*) is a unique function which is always positive and monotonically increases in strict sense, i.e. $x>y\Leftrightarrow f\left( x \right)>f(y)$. Assume $F$ is the set of all the substitution rates, and is also a totally ordered set. Then, *f*(*x*)is considered as an order-preserving mapping between *D* and *F*.

Assumption 2: Additivity hypothesis

We assume *f*(*x*) has additivity: *f*(*a* + *b*) = *f*(*a*) + *f*(*b*), where *a* and *b* is real numbers. This means *f*(*x*)is a bijective mapping.

Proof

Now, consider spatial sampling. The number of the sampling locations is *l*. Spatial sampling tries to select a combination of locations that minimizes the next function (Aoki & Ito 2020):

$$U\left( l \right)=\sum_{i=1}^{l} \sum_{j=1}^{l} e^{-d_{ij}}/\sqrt{d_{ij}}.$$

Although this function is defined for locations, this can simultaneously be considered to be defined for the set of all the distances, *D*. When the candidates of sampling locations are above the preset number of exploration, spatial sampling uses heuristics. Therefore, spatial sampling actually does not always minimize the above function. However, we assume that spatial sampling always minimizes it here. Now the subset *d* of *D* that minimizes the above function maximizes the next function:

$$U^{'}\left( l \right)=\sum_{i=1}^{l} \sum_{j=1}^{l} g\left( d_{ij} \right),$$

where $g\left( d_{ij} \right)=-e^{-d_{ij}}/\sqrt{d_{ij}}$. This function *ɡ*(*d_ij_*) monotonically increases in strict sense for *d_ij_* > 0, and this means *ɡ*(*x*) is an ordered-preserving mapping. Therefore, *f*{*ɡ*(*d_ij_*)} can be considered as the substitution rate. When *U’*(*l*) is maximized by *d*,

$$f\{\sum_{i=1}^{l} \sum_{j=1}^{l} g\left( d_{ij} \right)\}$$

is also maximized because *f*(*x*) monotonically increases or is an order-preserving mapping (Appendix S2; lemma). Also, because *f*(*x*) is assumed to have additivity and to be a bijection mapping,

$$f\{\sum_{i=1}^{l} \sum_{j=1}^{l} g\left( d_{ij} \right)\}=\sum_{i=1}^{l} \sum_{j=1}^{l} f\{g\left( d_{ij} \right)\}$$

is true. Now, the expected value of $\tilde{\varsigma}$ for *m* alleles in *l* locations is

$$E\left[ \tilde{\varsigma} \right]=E\left[ \frac{1}{m\left( m-1 \right)}\sum_{i=1}^{m} \sum_{j=1}^{m} \pi_{ij} \right]$$

$$=E\left[ \frac{1}{m\left( m-1 \right)}\sum_{i=1}^{m} \sum_{j=1}^{m} {[f\{g(d}_{ij})\}+\mu_{i}+\mu_{j}] \right]$$

$$=E\left[ \frac{1}{m\left( m-1 \right)}\sum_{i=1}^{m} \sum_{j=1}^{m} {f\{g(d}_{ij})\} \right]+E[\frac{1}{m\left( m-1 \right)}\sum_{i=1}^{m} \sum_{j=1}^{m} \mu_{i}+\mu_{j}].$$

Assuming random sampling within each location, the last term in the above equation equals to 0. Since *f*(*x*) monotonically increases in strict sense, different locations are always expected to have different alleles. Therefore, the summation for *m* alleles can be transformed to *l* locations. Thus,

$$E\left[ \tilde{\varsigma} \right]=\frac{1}{l\left( l-1 \right)}E\left[ \sum_{i=1}^{l} \sum_{j=1}^{l} {f\{g(d}_{ij})\} \right].$$

As shown above, $\sum_{i=1}^{l} \sum_{j=1}^{l} {f\{g(d}_{ij})\}$ is maximized under spatial sampling. Thus, $\tilde{\varsigma}$ is expected to be maximized and to be equal to ς*_l_*.$∎$

**Appendix S2. Lemma.**

Consider two totally ordered set *X* and *Y* with the size *N*, their *k*-subsets (a family composed of subsets with the number of *k* elements) *X’* and *Y’* and an order-preserving mapping *f*:*X’* → *Y’*. When the total sum of the elements $\sum x$ in a set $A\subset X'$ is the maximum among *X’*, $f(\sum x)$ is the maximum among the total sums of the elements of all the subsets in *Y’*.

Proof.

Arrange the elements in *X* as *x*_1_ ≤ *x*_2_ ≤ ⋯ ≤ *x_N_* according to the size order. When the total sum of the elements in $A$ is the maximum, the elements *x* in *A* are *x_N_*_-_*_k_*_+1_ ,*x_N_*_-_*_k_*_+2_, … , *x_N_* because *A* is also a totally ordered set. Now the elements composing $f(\sum x)$ are *k* larger elements in *Y* because *f*(*x*) is an order-preserving mapping. Thus, $f(\sum x)$ is the largest. $∎$
